# Action at a distance: organic cation induced long range organization of interfacial water enhances hydrogen evolution and oxidation kinetics†

**DOI:** 10.1039/d3sc03300g

**Published:** 2023-09-26

**Authors:** Kaiyue Zhao, Hao Yu, Haocheng Xiong, Qi Lu, Yi Qin Gao, Bingjun Xu

**Affiliations:** a College of Chemistry and Molecular Engineering, Peking University Beijing 100871 China b_xu@pku.edu.cn; b State Key Laboratory of Chemical Engineering, Department of Chemical Engineering, Tsinghua University Beijing 100084 China

## Abstract

Engineering efficient electrode–electrolyte interfaces for the hydrogen evolution and oxidation reactions (HOR/HER) is central to the growing hydrogen economy. Existing descriptors for HOR/HER catalysts focused on species that could directly impact the immediate micro-environment of surface-mediated reactions, such as the binding energies of adsorbates. In this work, we demonstrate that bulky organic cations, such as tetrapropyl ammonium, are able to induce a long-range structure of interfacial water molecules and enhance the HOR/HER kinetics even though they are located outside the outer Helmholtz plane. Through a combination of electrokinetic analysis, molecular dynamics and *in situ* spectroscopic investigations, we propose that the structure-making ability of bulky hydrophobic cations promotes the formation of hydrogen-bonded water chains connecting the electrode surface to the bulk electrolyte. In alkaline electrolytes, the HOR/HER involve the activation of interfacial water by donating or abstracting protons. The structural diffusion mechanism of protons in aqueous electrolytes enables water molecules and cations located at a distance from the electrode to influence surface-mediated reactions. The findings reported in this work highlight the prospect of leveraging the nonlocal mechanism to enhance electrocatalytic performance.

## Abstract 2

The structure-making ability of bulky hydrophobic cations promotes the formation of hydrogen-bonded water chains connecting the electrode surface to the bulk electrolyte, which in turn facilitates the hydrogen oxidation and evolution reactions.

## Graphical abstract

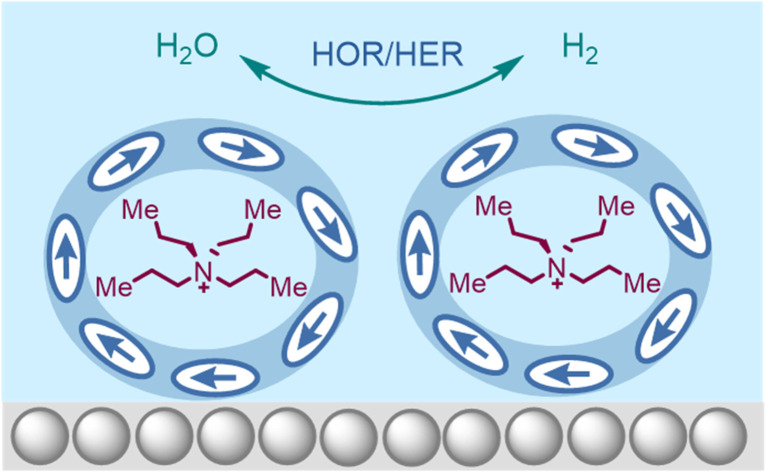

## Introduction

Efficient interconversion between the chemical and electrical forms of energy *via* electrocatalytic hydrogen evolution and oxidation reactions (HOR/HER) is a cornerstone for the green H_2_ economy. Research on the HOR/HER is fundamental towards the understanding of electrochemical reactions occurring on solid electrode surfaces.^1–4^ The central role of hydrogen in the future green energy landscape has afforded HOR/HER research renewed practical significance. In search for effective catalyst design strategies, literature discussion is often explicitly or implicitly centered around one question: is there a single descriptor capable of predicting the HOR/HER activity?^5–7^ The conventional view considers the electrocatalyst as the critical component, which is justified by the stark difference in activities among different electrode materials.^8,9^ The hydrogen binding energy (HBE) of the electrocatalysts was proposed as a predictor for the performance in the HOR/HER.^10–12^ A volcano-shaped plot of the HOR/HER activity *vs.* the HBE was reported with Pt possessing the optimal activity.^8^ The theory was challenged by the observation that the intrinsic HOR/HER activity remained dependent on the electrolyte pH on the reversible hydrogen electrode (RHE) scale,^10^ in contrast to the expected Nernstian behavior. The much lower HOR/HER rates in a base on precious metals pose a cost challenge for hydrogen fuel cells and water electrolyzers operating under alkaline conditions. Hydroxide binding energy (OHBE) has also been proposed as a relevant descriptor of the HOR/HER kinetics, especially on catalysts containing oxophilic metals.^13,14^ In addition, the nature of alkali metal cations, as well as the structure of near surface water under the influence of an interfacial electric field or adsorbed organic species, could also impact HOR/HER activities.^15–17^ Thus, multiple descriptors likely need to be taken into account in the design and optimization of electrochemical interfaces for efficient HOR/HER.

A common feature for all descriptors for the HOR/HER identified so far is that they can impact the micro-environment immediate to the surface-mediated reaction.^5^ The HBE and OHBE of catalysts could directly impact the stability of surface intermediates, while near surface water and cations could affect the energetics of surface bound species *via* short range interactions, *e.g.*, hydrogen bonds. Similarly, they could also influence the stability of the activated complex in the rate-determining step (RDS). Meanwhile, there is reason to believe that species that are outside the immediate micro-environment of surface reactions could also impact HOR/HER rates. HOR/HER involve shuttling protons to and from the electrode surface.^18–20^ Proton transfer occurs *via* structural diffusion, *i.e.*, the Grotthuss mechanism, which requires the activation of a number of water molecules adjacent to the proton being transported. Thus, the transfer of a proton from (or to) a water molecule to (or from) the electrode surface in the Volmer step in the HER (or the HOR), the likely RDS, could be impacted by water molecules a few hydrogen bonds away from the electrode surface. So far, no experimental evidence has emerged for this type of action-at-a-distance pathway to impact the HOR/HER, but recent computational results hinted at its plausibility.^21^

In this work, we report compelling experimental and computational evidence that such a nonlocal mechanism exerts a substantial impact on the HOR/HER kinetics at the Pt-electrolyte interface. HOR/HER kinetics were determined in a series of tetraalkyl ammonium hydroxide electrolytes and showed that bulky organic cations such as tetrapropyl ammonium (TPA) enhanced the exchange current density of the HOR/HER by more than a factor of 4 *vs.* that in KOH electrolyte with identical concentration. When both tetramethyl ammonium (TMA) and TPA coexist in the electrolyte, *in situ* surface enhanced infrared absorption spectroscopy (SEIRAS) shows that the less bulky TMA is preferentially located near the electrode surface with TPA further away from the interface. Strikingly, TPA is able to enhance the HOR/HER kinetics without directly interacting with intermediates and activated complexes in the surface mediated reaction. Molecular dynamics simulations and *in situ* spectroscopic results indicate that the structure-making TPA is able to enhance the formation and lifetime of hydrogen bonds among interfacial water molecules, especially along the surface normal direction. This effect persists even when TPA is located further away from the electrode surface than TMA, because the presence of TPA could foster the formation of chains of hydrogen-bonded water from the electrode surface to the bulk electrolyte. The impact of long-range organization of interfacial water on the HOR/HER kinetics highlights the nonlocal nature of electrode mediated reactions involving water activation. The proposed mechanism is further supported by control experiments, in which structure-breaking alkali metal cations effectively suppress the HOR/HER activity.

## Results

### Cyclic voltammetry and HOR/HER activity on Pt with tetraalkyl ammonium cations

**Fig. 1:**
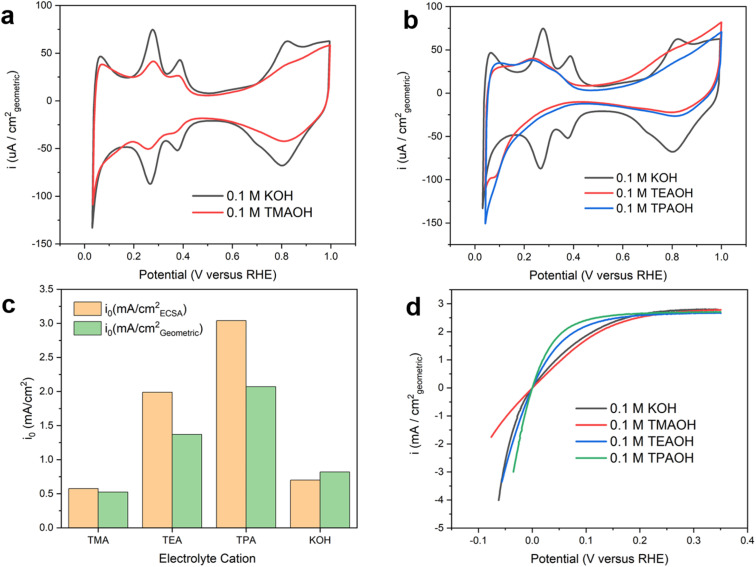
Cyclic voltammograms and HOR/HER activities of pc Pt. (a) CVs on pc Pt in Ar saturated 0.1 M KOH and 0.1 M TMAOH. (b) CVs on pc Pt in Ar saturated 0.1 M TEAOH and TPAOH. (c) Exchange current densities on pc Pt in H_2_ saturated 0.1 M KOH, 0.1 M TMAOH, TEAOH and TPAOH. (d) HOR/HER polarization curves on pc Pt in H_2_ saturated 0.1 M KOH, 0.1 M TMAOH, 0.1 M TEAOH and 0.1 M TPAOH.

The presence of quaternary ammonium cations significantly impacts the recorded cyclic voltammograms (CVs) on the polycrystalline Pt (pc-Pt) surface in a base. The CV collected on Pt in 0.1 M KOH exhibits well-defined underpotential deposited hydrogen (H_upd_) features (Fig. 1a), with peaks centered at 0.28 V and 0.38 V (all potentials reported in this work are referenced to the reversible hydrogen electrode, or RHE, unless noted otherwise) assigned to the deposition/oxidation of H_upd_ on the 110 and 100 facets of Pt.^22^ More recently, the more positive feature was interpreted as a displacement reaction between OH and H_upd_.^23^ Regardless of the interpretations, these CV features are intricately related and sensitive to the double layer structure, which could serve as a proxy to understand the impact of various species at the electrochemical interface on the structure and catalytic activity of the double layer. In 0.1 M tetramethylammonium hydroxide (TMAOH), the H_upd_ features are partially suppressed without any noticeable peak shift compared to those in 0.1 M KOH. In addition, the CV features of OH_ad_ deposition and reduction are also substantially suppressed in TMAOH (Fig. 1a). Distinct from TMAOH, with tetraethylammonium hydroxide (TEAOH) and tetrapropylammonium hydroxide (TPAOH), the H_upd_ features are mostly suppressed and only a fraction of broad H_upd_ features attributable to the Pt(111) facet remain. The preferential suppression of the H_upd_ features on the 110 and 100 facets could be attributed to the stronger non-covalent interaction between the tetraalkyl ammonium and the under-coordinated surface sites, possibly related to the Smoluchowski effect of stepped crystallographic facets.^24^ The steric interactions among large organic cations may also prevent them from forming a close packed layer and leave a fraction of surface sites accessible even at the saturation coverage of the organic adsorbate. Control experiments were conducted to rule out the possibility that impurities in the electrolytes cause changes in the recorded CVs and measured HOR/HER activities (ESI Note I and Fig. S1–5†). The suppression of H_upd_ features on Pt in the presence of organic cations has also been observed by other groups.^25–27^ Capacitive currents in the double layer region (0.5–0.7 V) are substantially smaller with larger organic cations than K^+^ (Fig. 1b), which is indicative of lower double layer capacitance. The more effective suppression of the H_upd_ features and reduction of double layer capacitance with bulkier organic cations could be attributed to the accumulation of hydrophobic organic cations in the double layer at the expense of interfacial water. Lower interfacial water density leads to reduced permittivity of the double layer and in turn lower capacitance. Although occasionally proposed in recent literature,^26,28^ we consider the specific adsorption of tetraalkyl ammonium cations on Pt by forming covalent bonds unlikely due to the chemical inertness of alkyl groups, which is in contrast with the theophylline derivatives reported in our recent work.^29^ CVs collected with TEA and TPA largely overlap, which could be because the impact of the enhanced hydrophobicity of cations with a longer alkyl chain is offset by their reduced concentration at the double layer due to the steric hindrance.

Intrinsic HOR/HER activity depends sensitively on the identity of cations, but does not correlate with the size of the H_upd_ features. Intrinsic HOR/HER activity on Pt, *i.e.*, the exchange current density, in the presence of different cations (pH = 13, Table S2†) was determined using the rotating disk electrode (RDE) method by fitting the polarization curves with the Butler–Volmer equation (Fig. S6†). Reported exchange current densities are normalized to the electrochemical surface area based on the amount of charge under H_upd_ features. Intriguingly, the HOR/HER activity grows with the size of organic cations from TMA to TPA (Fig. 1c). Compared to the exchange current density in 0.1 M KOH, the HOR/HER activity is slightly suppressed with TMA, while TEA and TPA enhance the intrinsic activity by a factor of 2.8 and 4.3, respectively. In terms of the exchange current density normalized with the geometric area, TEA and TPA enhance the HOR/HER activity by a factor of 1.7 and 2.5, respectively. Polarization curves with organic cations investigated in this work are stable during repeated potential scans (Fig. S7†).

**Fig. 2:**
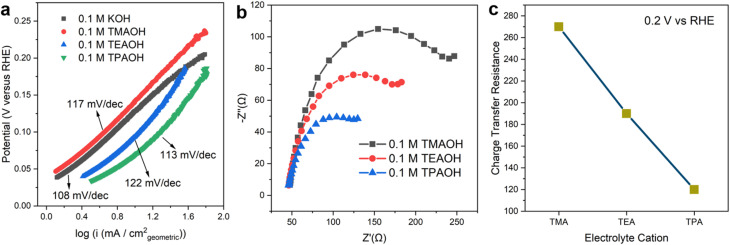
Tafel analysis and electrolyte dependent charge transfer resistance. (a) Tafel plots on pc Pt in Ar saturated 0.1 M KOH, 0.1 M TMAOH, 0.1 M TEAOH and 0.1 M TPAOH. (b) Nyquist plots of pc Pt at 0.2 V *vs.* RHE in Ar saturated 0.1 M TMAOH, TEAOH and TPAOH. (c) Fitted charge transfer resistance in Ar saturated 0.1 M TMAOH, TEAOH and TPAOH.

The identity of cations has a significant impact on the kinetics of the Volmer step. The Tafel slopes of the HOR branch are close to 120 mV dec^−1^ in all cations employed in this work (Fig. 2a), which is in line with previously reported values in the alkaline electrolyte on Pt.^22,30^ This observation indicates that the reaction follows the Tafel–Volmer mechanism with the Volmer step as the rate determining step (RDS).^30^ The comparable Tafel slope values provide a strong indication that the RDS remains unchanged in the presence of all organic cations investigated. Electrochemical impedance spectroscopy (EIS) was employed to understand the impact of different cations on the rate of the RDS. The obtained Nyquist plots were fitted with the equivalent electric circuit in Fig. S8.† The charge transfer resistance (*R*_ct_) reflects the energy barrier of heterogeneous hydrogen adsorption/desorption reactions on Pt in a base, *i.e.*, H_2_O + e^−^ ↔ H_ad_ + OH^−^. The size of semicircles in the Nyquist plots (Fig. 2b) is inversely correlated to *R*_ct_. The size of the semi-circle decreases as the size of organic cations increases from TMA to TPA, suggesting that larger cations facilitate the charge transfer in hydrogen adsorption/desorption in the H_upd_ region (Fig. 2c). It can be inferred that larger organic cations enhance the HOR/HER activities through accelerating the Volmer step.

### Role of the interfacial electric field in the HOR/HER

The vibrational Stark effect of adsorbed CO on Pt was leveraged to investigate the effect of the identity of organic cations on the interfacial electric field. According to the classic Gouy–Chapman–Stern (GCS) model of the electric double layer, potential change across the electrochemical interface mostly occurs between the electrode surface and ions residing in the outer Helmholtz plane (OHP) in concentrated electrolytes (concentration ≥0.1 M), *i.e.*, the double layer can be reasonably modeled as a planar capacitor (by neglecting the potential drop across the diffuse layer).^31^ It follows that the potential difference between the electrode and the OHP is the same at a constant electrode potential, and thus the interfacial electric field strength depends only on the distance between the electrode surface and the OHP (*d*_H_). Bulkier cations (below the potential of zero charge (PZC)) are expected to lead to larger *d*_H_ due to steric hindrance, and in turn weaker interfacial field strength. The interfacial field strength was proposed to have a significant impact on the rigidity of the interfacial water network during the HOR/HER.^32,33^ A weaker interfacial electric field leads to a lower energy barrier necessary in the reorganization of interfacial water to transport the proton/hydroxide through the double layer and accelerate the reaction.^34^ Although determining the absolute strength of the electric field strength across the electrochemical interface is challenging, the relative interfacial electric field strength among different cations could be compared by the Stark shift of a specifically adsorbed probe molecule such as CO.^35,36^ The extent of shift in the peak wavenumber per unit shift of potential, referred to as the electrochemical Stark shift in this work, provides an experimentally accessible measure of the interfacial electric field strength (Table 1). The greater the electrochemical Stark shift value, the stronger the interfacial electric field is. Control experiments show that CO coverage on Pt is largely independent of the identity of cations (Fig. S9†), so the electrochemical Stark shift is unlikely caused by the coverage effect, *i.e.*, the dynamical coupling effect.^37^ The electrochemical Stark shift decreases as the size of the organic cation grows from TMA to TPA (Table 1), which is consistent with the expectation that larger cations lead to greater *d*_H_ and the lower strength of the interfacial electric field. Although the introduction of a strongly adsorbing probe molecule, such as CO, on Pt could substantially impact the double layer structure, the relative impact of different organic cations with similar structures, *e.g.*, tetra-alkyl ammonium with different lengths of the alkyl chains, on the interfacial field strength is not expected to be affected by the adsorbed CO. This is because the interaction between tetra-alkyl ammonium and the electrode is largely electrostatic in nature without covalent bonding, which is relatively insensitive to the presence of the interfacial adsorbed CO layer.

**Table 1:** Cation-dependent electrochemical Stark shift in 0.1 M hydroxide solutions

Electrolyte cation	Electrochemical Stark shift (cm^−1^ V^−1^)	Exchange current density (mA cm_ECSA_^−2^)
0.1 M TMA	36.5 ± 1.0	0.54 ± 0.08
0.1 M TEA	31.9 ± 0.9	1.97 ± 0.03
0.1 M TPA	24.9 ± 1.7	3.05 ± 0.06
99 mM TPA + 1 mM TMA	30.8 ± 1.2	2.78
98 mM TPA + 2 mM TMA	32.0 ± 0.7	2.66
90 mM TPA + 10 mM TMA	36.2 ± 0.9	1.56 ± 0.12
50 mM TPA + 50 mM TMA	36.3 ± 0.8	1.52 ± 0.07

**Fig. 3:**
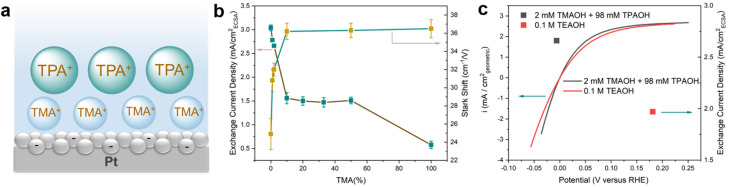
HOR/HER activities of pc Pt in the mixture of TMA and TPA. (a) Schematic of the distribution of cations when the concentration of TMA is higher than 0.01 M in the mixture of TMA and TPA. (b and c) HOR/HER activities and electrochemical Stark shifts on pc Pt in H_2_ saturated 0.1 M hydroxide solutions with mixed TMA and TPA.

The correlation between the electrochemical Stark shift and the HOR/HER activity is far from straightforward. The HOR/HER activity increases from TMA to TPA, which is in line with the declining electrochemical Stark shift (36.5 to 24.9 cm^−1^ V^−1^) and weakening electric field strength. However, the correlation between the electric field strength and the HOR/HER activity fails to hold when a mixture of TMA and TPA is used while maintaining the overall cation concentration at 0.1 M. Introducing 1 mM TMA into TPA (99 mM) leads to an increase in the electrochemical Stark shift of 5.9 cm^−1^ V^−1^ to 30.8 cm^−1^ V^−1^ from that in 0.1 M TPA (Table 1 and Fig. S10†). It appears that TMA is enriched at the double layer (Fig. 3a) and plays a decisive role in determining the interfacial filed strength. This is likely because that TMA could get closer to the negatively charged electrode surface than the bulkier TPA, and thus is energetically more favorable to be located at the double layer. The presence of TMA reduces *d*_H_ and in turn increases the electrochemical Stark shift. Importantly, the significant reduction in the electrochemical Stark shift (and the field strength) with 1 and 2 mM TMA in the electrolyte is not accompanied by a comparable decrease in the exchange current density of the HOR/HER (Fig. 3b and Table 1). The electrochemical Stark shift of 2 mM TMA and 98 mM TPA (32.0 cm^−1^ V^−1^) is comparable to that of 0.1 M TEA (31.9 cm^−1^ V^−1^), but the exchange current density of the latter is lower by more than 20% (Fig. 3c). These results indicate that the interfacial electric field strength is not a sensitive indicator of the HOR/HER activity in the current system. Furthermore, the electrochemical Stark shift in the electrolyte containing ≥10 mM TMA (with balancing TPA) is experimentally indistinguishable from that of 0.1 M TMA (Table 1); however, the HOR/HER activity decreases monotonically with the increase in the TMA concentration (Fig. 3b and Table 1). In particular, the exchange current density in 0.1 M TMA is only about 1/3 of that in 0.05 M TPA + 0.05 M TMA (Table 1). This set of data raise an intriguing question: since the OHP is primarily occupied by TMA (as indicated by the constant electrochemical Stark shift at ≥10 mM TMA), how does TPA influence the rate of reaction occurring on the electrode surface from a distance? The mechanism through which TPA influences the electrode surface mediated reaction at a distance is investigated *via* molecular dynamics (MD) simulations in the next section.

### Molecular dynamics simulations

**Fig. 4:**
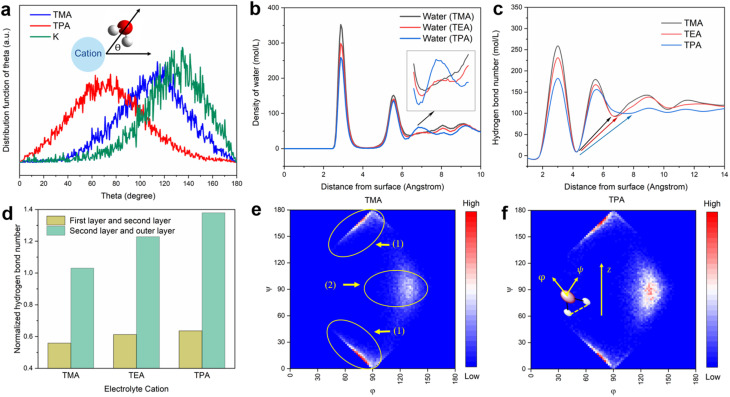
MD simulations of the hydrogen bonding structure. (a) Distribution functions of *θ* of K^+^, TMA and TPA. *θ* is the angle of the water dipole relative to the direction of the electric field generated by a cation, as shown in the inset. (b) Distribution functions of water molecules with TMA, TEA and TPA as electrolyte cations along the direction of surface normal. (c) Distribution of the number of hydrogen bonds along the direction of surface normal. (d) Average numbers of hydrogen bonds between different water layers. Heat map of (*φ*, *ψ*) distribution of adsorbed water molecules at (e) Pt/TMA interface and (f) Pt/TPA interface. *φ* is the angle between the water dipole and the surface normal, while *ψ* is the angle between the normal of the plane of the water molecule and the surface normal, as shown in the inset of (f). Position 1 represents equatorial water molecules, where O–H bonds are largely parallel to the electrode surface. Position 2 represents water molecules with O–H bonds along the surface normal. Results in (b)–(f) were simulated at a potential of −0.1 V.

MD simulations of the electrochemical interface reveal that the identity of cations indeed has a significant impact on the structure of the surrounding water. The distribution of the angle (*θ*) between the water dipole vector (defined as from the positive to the negative end) and the electric field generated by the adjacent cation (K^+^, TMA, and TPA) were first computed with a 4 Å cut-off radius from the cations (Fig. 4a). The most probable *θ* for K^+^ is ∼140°, suggesting that the water dipole in the hydration level is close to being anti-parallel with the electric field vector. This water dipole orientation is expected as the strong electrostatic interaction between water and K^+^ forces adjacent water molecules to break away from its hydrogen-bond network of bulk water, *i.e.*, the structure-breaking effect.^38^ In contrast, the most probable *θ* for TPA is ∼80°, indicating that the water dipole is primarily normal of the electric field. This latter observation can be attributed to the reduced electric field strength due to the diffusive charge and the hydrophobic alkyl groups. To compensate for the loss of coordination with cations, surrounding water molecules tend to respond by forming an extensive hydrogen-bond network, *i.e.*, the structure-making effect.^38^ Such an interpretation is supported by the higher average number of hydrogen bonds of water molecules surrounding TPA than K^+^ (Table S1†). As expected, the values of both *θ* and average number of hydrogen bonds for TMA are in between those for TPA and K^+^.

To further probe how near-surface organic cations impact the physical properties of the interfacial water structure, we analyzed the MD trajectories obtained for the Pt(111)-aqueous interface with different organic cations as the electrolyte. Representative snapshots of the Pt/electrolyte interface show that organic cations are able to displace a substantial fraction of interfacial water, which is likely the cause for the reduced H_upd_ features in CVs (Fig. 1a and b). Maxima in the plot of the density of water molecules (Fig. 4b) and the number of hydrogen bonds (Fig. 4c) *vs.* the distance from the electrode surface correspond to layers of water from the surface.^21^ Both the density of water and the number of hydrogen bonds of the first layer of water (at 2.9 Å) follow the order of TMA > TEA > TPA, indicating that bulkier organic cations are more effective in displacing adsorbed water on Pt. Importantly, the feature at a distance of about 7 Å from the surface grows in intensity with the size of organic cations (inset of Fig. 4b). Similar features were also observed on the Pt(100) facet, suggesting that the distribution of interfacial water was largely facet independent (Fig. S11†). Since this feature is located in between the 2nd and 3rd layers of water, the strengthening of this feature indicates a stronger connection among water molecules *via* hydrogen bonds from these layers in the presence of bulkier cations. This interpretation is supported by the increasingly uniform distribution of the number of hydrogen bonds in the presence of larger organic cations (indicated by arrows in Fig. 4c). The normalized number of hydrogen bonds (on the per organic cation basis) between the first two layers of water, as well as that between the 2nd and outer layers of water, increases from TMA to TPA (Fig. 4d), further supporting bulky organic cations' ability to promote interlayer connections. The orientation distribution of the 1st layer of water molecules from the surface was also determined to understand the impact of the nature of cations on the interfacial water structure. *φ* and *ψ* are defined as the angle between the water dipole and the surface normal, and the angle between the surface normal of a water molecule and the surface normal of the electrode, respectively (inset of Fig. 4f). For example, the (0, 90) points in Fig. 4e and f refer to water molecules with the dipole parallel to the surface normal, and the points (90, 0) and (90, 180) refer to water molecules with their planes parallel to the surface (Fig. S12a and b†). Comparing the orientation distribution of interfacial water molecules with TMA and TPA, the most pronounced difference is the higher fraction of water in the configuration around the (130, 90) point (schematically shown in Fig. S12c†) of the latter than the former. This configuration corresponds to the formation of interlayer, rather than intralayer, hydrogen bonds. Collectively, these results indicate that TPA promotes connection between different layers of interfacial water, while TMA favors intralayer hydrogen bonds. Since the HOR/HER involves shuttling of protons between the interface and the bulk electrolyte, interconnection between different layers of water at the interface is crucial.^21^ Water exists in structured layers only at the electrified interface and the layered structure tends to smooth out as the distance from the electrode surface increases (Fig. 4b). Cations capable of facilitating interlayer connection at the interface *via* hydrogen bonds have been shown to promote the HOR/HER activity.^21^ Although the MD method employed in this work is optimized for interactions among species in the electrolyte and cannot take the effect of surface adsorbed H on the double level structure into account, existing *ab initio* MD simulations show that adsorbed H on Pt(111) increases the distance between the first layer of water and the electrode surface and has a negligible impact on the configuration of water molecules beyond the first layer.^39^ Since the focus of this work is on the effect of interfacial water molecules beyond the OHP on the electrode surface-mediated reaction, it is reasonable to believe that the computational results presented above are able to capture the key features of the long range interactions among interfacial water molecules.

**Fig. 5:**
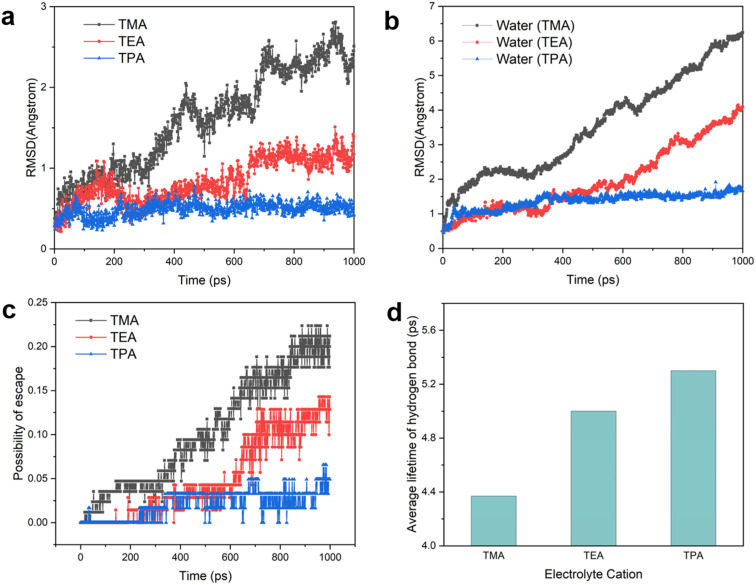
Dynamics of near-surface cations, water molecules and hydrogen bonds. (a) Root-mean-square displacement of cations. (b) Root-mean-square displacement of water molecules in the presence of different cations. (c) Possibility of escape of water from the Pt(111) surface. (d) Extracted lifetime of hydrogen bonds from time autocorrelation functions.

Having established the structure of the hydrogen bonding network of interfacial water, we turn to the dynamic behaviors of interfacial species. The root-mean-square displacement (RMSD) of organic cations, with which the diffusion coefficient can be derived, provides a measure of the mobility of cations at the interface. The RMSD decreases in the following order: TMA > TEA > TPA (Fig. 5a), which may be explained by the stronger van der Waals force between the Pt electrode and bulkier cations.^26^ Since interfacial water molecules organize to a significant extent based on their interaction with adjacent cations (when the electrode is negatively charged), the mobility of cations is expected to impact the mobility of water molecules around them. This prediction is the same as that of the calculated RMSD of water: TMA > TEA > TPA (Fig. 5b). Consistent with this trend, at a given time interval, the probability of water molecules escaping from the layer closest to the Pt surface also decreases with the increase in the size of organic cations (Fig. 5c). It is established that the rate of concerted proton hops is dependent on the stability of the hydrogen bond network, known as the Grotthuss mechanism.^40^ More stable hydrogen bonds in the network lead to higher rates of proton transport. The lifetimes of hydrogen bonds with different organic cations were extracted *via* the time autocorrelation function of hydrogen bonds (see the Methods and Fig. S13† for details). The average lifetime of hydrogen bonds varies in the opposite trend as the cation and water mobility, *i.e.*, TPA > TEA > TMA (Fig. 5d), as the diffusion of both cations and water molecules necessarily causes breaking of hydrogen bonds. Thus, bulkier cations such as TPA capable of stabilizing hydrogen bonds in interfacial water molecules are expected to enhance the rate of proton shuttling *via* the Grotthuss mechanism.

**Fig. 6:**
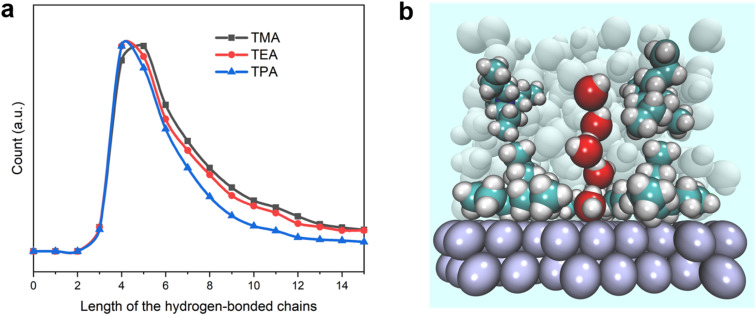
Cation-dependent hydrogen-bonded water chains at the electrified Pt(111) surface. (a) Statistical numbers of hydrogen bond chains of different lengths between the water molecules within 4.4 Å and beyond 10.4 Å. The distance in the *xy* plane between the start and end points was constrained within 3 Å. (b) Snapshot of the hydrogen bond chain around TPA.

To further confirm the enhanced connectivity among layers of interfacial water and increased stability of hydrogen bonds in the presence of bulkier organic cations, the distribution function for the spatial dimension of the hydrogen bond network within the interfacial water was evaluated. The distribution of the length of hydrogen-bonded chains connecting the 1st layer of water from the surface (≤4.4 Å) to water molecules outside the 3rd layer (≥10.4 Å) was determined (details of the counting approach are included in the Methods section). The most probable number of water molecules in the hydrogen-bonded chains with both TEA and TPA is 4, while it is 5 for TMA (Fig. 6a). The full width at half maximum (FWHM) of the distribution decreases in the sequence of TPA < TEA < TMA. Thus, the connection between water molecules residing in different layers is most direct in the presence of TPA, followed by TEA, and then TMA. This observation is consistent with the higher degree of interlayer connectivity of water in the presence of bulkier cations (Fig. 4b and c). More direct connection *via* fewer hydrogen-bonded water molecules makes the breaking of the water chain less likely because each additional hydrogen bond in the water chain increases the likelihood of at least one or hydrogen bonds breaking at a given moment. The combination of a longer lifetime and more direct hydrogen-bonded water channel along the surface normal direction with TPA is expected to facilitate the proton shuttling critical to the HOR/HER rates, which is consistent with the enhanced exchange current density in the presence of TPA (Fig. 1c).

The ability of TPA to direct the hydrogen bonded chains along the surface normal direction and enhance the lifetime of hydrogen bonds among surrounding water could help rationalize TPA's ability to enhance the HOR/HER rates even though it is not located at the OHP. Regardless of whether the hydrogen-bonded water molecules are located within the OHP or the diffuse layer, as long as they are in the path enabling the proton shuttling, their orientation and stability are expected to have an impact on the HOR/HER activity. Even though TPA is further away from the electrode surface than TMA when they are both present, it still has a pronounced beneficial effect on the measured rates. In this sense, the general assumption that only species capable of impacting the immediate micro-environment of a reaction can influence its rates needed to be applied with caution. Furthermore, the presence of TPA adjacent to the TMA layer at the OHP is capable of reducing the mobility of TMA *via* van der Waals interaction and spatial confinement, leading to a higher lifetime of hydrogen bonds in the TMA/TPA system (Fig. S14†).

### Spectroscopic evidence of cations' impact on interfacial water

**Fig. 7:**
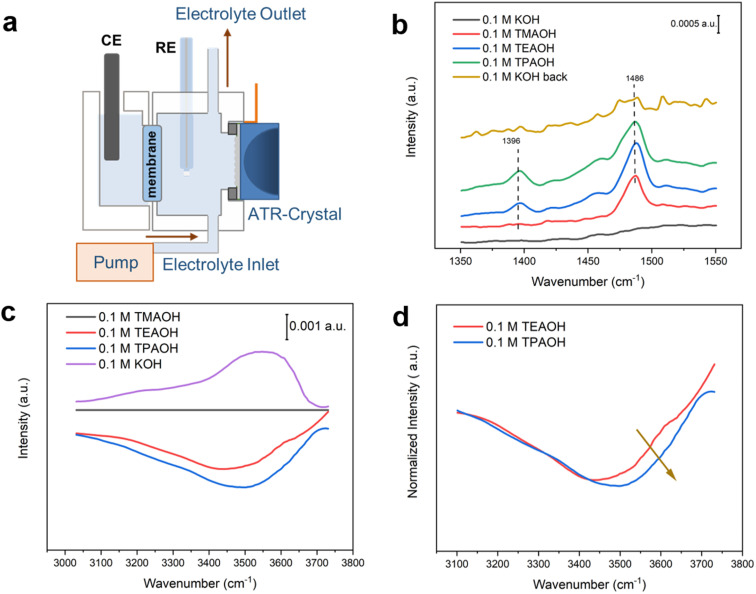
Spectroscopic analysis of the cation-dependent water structure on the Pt film electrode. (a) Schematic of the custom-designed SEIRAS flow cell. SEIRA spectra of C–H bending mode (b) and O–H stretching mode (c and d) in Ar saturated 0.1 M hydroxide solution with different cations at 0.5 V *vs.* RHE.

*In situ* surface enhanced infrared absorption spectroscopy (SEIRAS) investigations provide experimental evidence supporting the hypothesis proposed based on MD simulations. Highly hydrogen-bonded and under-coordinated water exhibit distinct features in vibrational spectroscopy, *i.e.*, the former corresponds to features at lower wavenumbers (3100–3400 cm^−1^),^17,41,42^ while the latter is responsible for higher wavenumber bands at 3500–3700 cm^−1^.^41,43^ A custom-designed SEIRAS flow cell was employed to allow for direct comparison of spectra collected in the different cations by quickly switching between electrolytes (Fig. 7a). No discernible feature is present in the spectrum collected in 0.1 M KOH at 0.5 V (in the double layer region) in the lower wavenumber range (Fig. 7b), indicating the lack of organic species. When the electrolyte was switched to 0.1 M TMAOH, a band appeared at ∼1486 cm^−1^, which could be attributed to the C–H bending mode of CH_3_.^27,28^ In addition to the 1486 cm^−1^ band, another band at ∼1396 cm^−1^ corresponding to the C–H bending mode of CH_2_ appeared when the electrolyte was switched to 0.1 M TEAOH. Similar spectral features were observed with TEA and TPA as they all possessed CH_2_ and CH_3_ groups. Switching the electrolyte back to KOH led to the disappearance of bands, suggesting that organic cations do not interact strongly with the Pt surface and can be completely displaced by switching the electrolyte in the flow cell. The impact of cations on the speciation of interfacial water is monitored by analyzing the broad band at 3100–3700 cm^−1^ corresponding to the O–H stretching mode of water (Fig. 7c). The spectrum collected in 0.1 M TMAOH is used as the reference. A prominent negative peak appears in bulkier cations, with the intensity growing with the size of the cations. This spectral observation reflects the increasing amount of interfacial water displaced by cations, following the order: TMA < TEA < TPA, consistent with the results of MD simulations (Fig. 4b). Importantly, a growing fraction of the negative band is in the spectral range of 3500–3700 cm^−1^ with bulkier cations. This trend is clearer when scaling the spectra to make their lower wavenumber regions largely overlap (Fig. 7d), which shows that a growing fraction of the higher wavenumber band (3500–3700 cm^−1^) is lost in the presence of bulkier organic cations. Since the higher wavenumber portion of the broad O–H stretching band has been attributed to weakly hydrogen-bonded water,^41^ these spectral observations are consistent with the MD simulations that a higher fraction of water molecules around bulkier organic cations tend to form hydrogen-bonded chains (Fig. 4 and 6). Furthermore, this conclusion is in line with previous studies on the water structure around tetraalkyl ammonium cations with other techniques, including X-ray^44^ and neuron diffraction^45^ and Raman scattering.^46^ In contrast, a band at ∼3550 cm^−1^ corresponding to weakly hydrogen-bonded water is present in 0.1 M KOH, indicating decreased hydrogen bonding among water molecules due to the structure breaking effect of K^+^.^38^

### Cation–water interactions

**Fig. 8:**
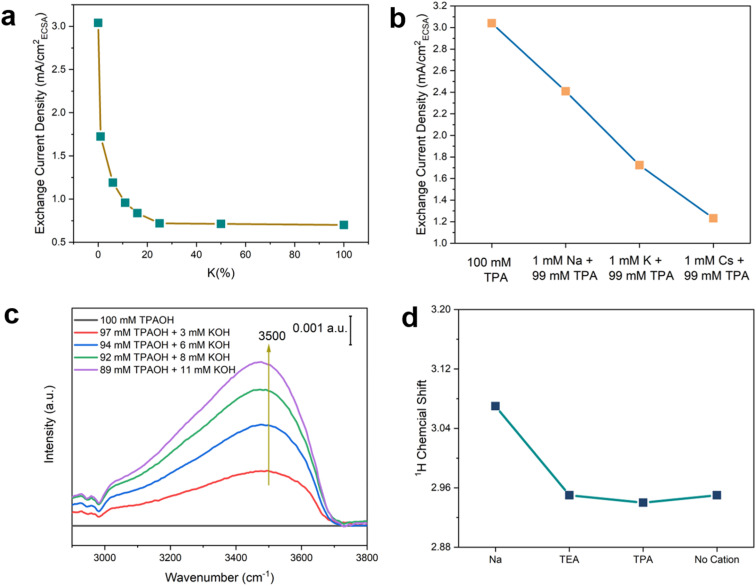
Effect of cation–water interaction. (a) HOR/HER activities in Ar saturated 0.1 M hydroxide solution with various ratios of K^+^ and TPA. (b) HOR/HER activities in Ar saturated 0.1 M hydroxide solution with 0.099 M TPA and 1 mM alkali metal cation, *i.e.*, Na^+^, K^+^, or Cs^+^. (c) SEIRA spectra of the O–H stretching mode in Ar saturated 0.1 M hydroxide solution with different ratios of TPA and K^+^ at 0.5 V *vs.* RHE. The reference spectrum was obtained in 0.1 M TPAOH. (d) ^1^H chemical shift of water with alkaline metal cations and organic cations (0.1 M cations and ClO_4_^−^ in acetonitrile with 1% water (mass fraction)).

To further test the hypothesis that the hydrogen-bonded chains of water around the hydrophobic bulky organic cations enhance the HOR/HER kinetics, the effect of structure-breaking alkali metal cations on the HOR/HER rate was determined. Alkali metal cations are known to disrupt their adjacent hydrogen bond networks *via* strong electrostatic interaction with the water dipole, and the structure-breaking effect increases in the sequence of Na^+^ < K^+^ < Cs^+^.^38^ Thus, the introduction of alkali metal cations into the TPA-containing electrolyte is expected to disrupt the hydrogen-bonded chains of water and reduce HOR/HER activities according to our hypothesis. Indeed, the introduction of 1% K^+^ (1 mM K^+^ + 99 mM TPA) leads to a steep drop in the exchange current density in the HOR/HER (by 43%) compared with 0.1 M TPAOH (Fig. 8a). The decline in the HOR/HER activity slows with K^+^ accounting for an increasing fraction of cations in the electrolyte (Fig. 8a). The exchange current density in K^+^/TPA electrolyte becomes indistinguishable from that of 0.1 KOH when K^+^% is above 20%. The exchange-current density decreases in the presence of 1 mM alkali metal cations and 99 mM TPA in the sequence of Na^+^ > K^+^ > Cs^+^ (Fig. 8b), indicating that the HOR/HER activity is inversely correlated with the structure-breaking ability of alkali metal cations.^38^ This trend is consistent with the reported sequence of HOR/HER activity in different alkali metal cations: Li^+^ > Na^+^ > K^+^ > Cs^+^,^47^ and is in line with the proposed beneficial role of interfacial hydrogen-bonded chains of water in the HOR/HER. To monitor the change of the interfacial structure upon introducing K^+^ into 0.1 M TPAOH, we performed *in situ* SEIRAS experiments with the spectrum obtained in 0.1 M TPAOH as the reference. A prominent peak centered at 3500 cm^−1^ appeared when 3 mM K^+^ was introduced into the electrolyte, and its intensity increased with the concentration of K^+^ (Fig. 8c). This band corresponds to the weakly hydrogen-bonded water.^41,43^ Concomitantly, the bands of C–H stretching (2900–3000 cm^−1^) and bending (1360–1520 cm^−1^) modes decrease simultaneously (Fig. 8c and S15†). Taken together, it can be inferred that the presence of a low concentration of K^+^ effectively disrupts the hydrogen-bonded water around TPA, impairing the mechanism through which TPA promotes the surface-mediated HOR/HER.

Nuclear magnetic resonance (NMR) spectroscopy was employed to provide additional evidence for the impact of cations on water. ^1^H-NMR spectra were collected in 0.1 M perchlorate in acetonitrile with 1 wt% water. The high cation-to-water ratio in the electrolyte was to enhance the effect of cations on water. The ^1^H chemical shift of water with Na^+^ (3.07 ppm) is significantly higher than that with organic cations (TEA and TPA), all of which appear at 2.95 ppm.^3^ TMA and K^+^ were not used due to the low solubility of the corresponding perchlorate salts in acetonitrile. Since chemical shifts to a low field indicate a lower electronic density of protons, the NMR results suggest that water coordinates more strongly with Na^+^ than organic cations, most likely due to the Lewis acidity of the former, leading to the polarization of the O–H bond in water. The chemical shift of water (1 wt% in acetonitrile) in the absence of cations is similar to that with organic cations, further confirming the weak interaction between water and organic cations. Alkyl groups are expected to effectively shield the positive charge of organic cations and weaken electrostatic interaction between the dipole of water and cations. The NMR results are consistent with the structure-breaking effect of alkali metal cations (except for Li^+^ due to its tight 1st hydration shell) and structure-making effect of tetraalkyl ammonium cations.

## Discussion

The HOR and HER involve not only the surface-mediated electron transfer to or from H, but also the shuttling of protons to and from the electrode surface. The chemical inertness of the alkyl groups in tetraalkyl ammonium cations makes direct chemical interaction on the surface mediated reaction unlikely. Proton shuttling in water occurs *via* structural diffusion, or the Grotthuss mechanism, which involves a number of hydrogen-bonded water molecules.^19–21,48^ In this regard, the HOR/HER activity depends not only on the surface mediated reaction, but also on the capacity in activating interfacial water for proton shuttling. Our molecular dynamics simulations show that the presence of structure-making organic cations makes hydrogen bonds among interfacial water more stable and more directional (normal to the electrode surface). We propose that organic cations, *e.g.*, TPA, enhance HOR/HER activity on pc Pt by facilitating the proton shuttling across the double layer. Since the proton shuttling occurs with the assistance of chains of hydrogen-bonded water,^20,48^ the long-range organization of interfacial water becomes an enabling feature. In electrolytes with both TMA and TPA, even though TMA is preferentially located next to the electrode surface, the presence of TPA could still exert its influence on the HOR/HER activity by stabilizing the hydrogen-bonded water chains. This interpretation is further supported by the detrimental effect of structure-breaking alkali metal cations, even at low concentrations relative to coexisting organic cations (Fig. 8a and b), on the HOR/HER activity.

It is important to note that the chains of hydrogen-bonded interfacial water do not enhance the HOR/HER by enhancing the mass transport of protons from or to the interface. If this is the case, the RDS would shift from the Volmer step in a TPAOH electrolyte to mass transport of protons in TMAOH. The similar Tafel slopes determined in all electrolytes investigated in this work (Fig. 2a) show that the Volmer step is the RDS regardless of the electrolyte. The kinetic barrier for extracting a proton from the interfacial water *via* its reduction in the HER, as well as donating a proton to the interfacial water in the HOR, depends on the structure of interfacial water. Extended chains of hydrogen-bonded interfacial water molecules likely facilitate the HOR/HER by stabilizing the transition state in the proton transfer step. The non-local impact of TPA on the HOR/HER reflects that the donor and acceptor in the proton transfer during the HOR/HER are not individual interfacial water molecules, but these hydrogen-bonded water chains extend a few molecular layers away from the catalyst surface. It follows that any interfacial species capable of impacting the stability of these water chains would affect the HOR/HER rate. Thus, interfacial water structure engineering should be taken into account in the design and optimization of electrolytes and electrochemical interfaces in H_2_ fuel cells and water electrolyzers.^49^

## Conclusions

In summary, we demonstrated that HOR/HER activities on pc Pt could be substantially impacted by the nature of cations, with structure-making organic cations such as TPA promoting the reaction while structure breaking alkali metal cations suppressing the activity. When both TMA and TPA are present in the electrolyte, TMA is preferentially located next to the electrode surface. Intriguingly, even though TPA is located relatively far away from the electrode, its presence could still enhance the HOR/HER activity. Molecular dynamics simulations show that the presence of bulky organic cations could stabilize hydrogen bonds among interfacial water molecules, and make these hydrogen-bonded water chains preferentially oriented along the surface normal direction. The ability of TPA to induce a long-range hydrogen-bonded water structure is proposed to be central to its beneficial effect on the HOR/HER kinetics and its capacity of acting at a distance. This hypothesis is supported by *in situ* SEIRAS results, which show preferential diminishment of weakly hydrogen-bonded water in the presence of TPA. The introduction of low concentrations of structure-breaking K^+^ into the TPA containing electrolyte leads to the formation of weakly hydrogen-bonded water and diminished HOR/HER activity. Our findings provide compelling evidence that long-range hydrogen-bonded interfacial water promotes the HOR/HER by facilitating the proton shuttling to and from the electrode surface and point to a promising strategy of enhancing electrocatalytic activities through interfacial water structure engineering.

## Data availability

All supporting data is provided in the ESI.†

## Author contributions

Bingjun Xu and Kaiyue Zhao conceived this project. Kaiyue Zhao and Haocheng Xiong carried out the experiments. Hao Yu and Yi Qin Gao performed the simulations. All the authors contributed to the final version of the manuscript.

## Conflicts of interest

There are no conflicts to declare.

## Supplementary Material

SC-014-D3SC03300G-s001
