# Supplementary material for: Action at a distance: organic cation induced long range organization of interfacial water enhances hydrogen evolution and oxidation kinetics
Source: Chem Sci. 2023 Sep 26;14(40):11076–87. doi: 10.1039/d3sc03300g (PMC10583708; doi:10.1039/d3sc03300g)
Supplement: SC-014-D3SC03300G-s001 [file SC-014-D3SC03300G-s001.pdf]

Electronic Supplementary Material (ESI) for Chemical Science.
This journal is © The Royal Society of Chemistry 2023

Supplementary information for

Action at A Distance: Organic Cation Induced Long Range Organization of Interfacial Water Enhances Hydrogen Evolution and Oxidation Kinetics

Kaiyue Zhao[1], Hao Yu[1], Haocheng Xiong [1,2], Yi Qin Gao[1], Qi Lu[2] and Bingjun Xu[1,*]

[1]College of Chemistry and Molecular Engineering, Peking University, Beijing 100871, China
[2]State Key Laboratory of Chemical Engineering, Department of Chemical Engineering, Tsinghua University, Beijing 100084, China

*Corresponding author: b_xu@pku.edu.cn

Materials

Graphite rod (99.995% trace metals basis) and potassium hydroxide (99.99%, trace metal basis) were purchased from Sigma-Aldrich. Pt foil (99.99% metals basis) and tetramethylammonium hydroxide (25% aqueous solution) were purchased from Alfa Aesar. Tetraethylammonium hydroxide (35% aqueous solution) and tetrapropylammonium hydroxide (40% aqueous solution) were all purchased from TCI. Hydrogen (99.999%), and argon (99.999%) were purchased from Air Liquide. Milli-Q water (18.2 MΩ cm) was used to prepare all the electrolytes.

Methods

Electrochemical measurements

All electrochemical measurements were conducted with a Gamry Interface 1000 Potentiostat in a homemade Teflon cell to circumvent the contamination from the corrosion of glass. A graphite rod was used as the counter electrode. A saturated calomel electrode (BASi) was used as the reference electrode. A 5 mm polycrystalline Pt rotating disk electrode (RDE) (Pine Instruments) was employed for electrochemical measurements. Before each experiment, the Pt disk was polished manually by 0.05 μm Al_2O_3 suspension and rinsed with Milli-Q water. The Pt electrode was subsequently electrochemically cleaned by cycling potential between 0.05 and 1.2 V vs RHE at a scan rate of 50 mV/s until reproducible cyclic voltammograms were achieved. All potentials reported in this work were corrected for the solution resistance (iR drop) before further analysis and referenced to the reversible hydrogen electrode (RHE).

Before any electrochemical tests, the homemade Teflon cell was boiled in Milli-Q water for at least 3 h. The solution resistance was determined by potentiostatic electrochemical impedance spectroscopy. Cyclic voltammograms were measured in Ar saturated 0.1 M hydroxide solution with different cations by cycling between 0.05-1.0 V vs RHE at a scan rate of 50 mV/s. HER/HOR polarization curves were obtained with a potential range of -0.1-0.5 V vs RHE in H_2 saturated electrolyte at a rotation rate of 1600 rpm. All HER/HOR polarization curves were normalized by the geometric area. The exchange current density was normalized by the electrochemical surface area (ECSA), which was calculated by the integrated area of the underpotential deposition hydrogen in CV with the assumption of 210 μC/cm^2 for the adsorption of one hydrogen monolayer on Pt. Kinetic current density for HOR was obtained using the Koutecky-Levich equation to remove the contribution of mass transport to the measured current density:

$$1/i = 1/i_{lim} + 1/i_k$$

Where i is the measured current density, i_{lim} is the limiting current density, and i_k is the kinetic current density.

The kinetic current density curves were fitted to the Butler–Volmer equation to get the exchange current density i_0.

$$i_k = i_0 \left(\mathrm{e}^{\frac{\alpha\eta F}{RT}} - \mathrm{e}^{-\frac{(1-\alpha)\,\eta F}{RT}} \right)$$

where η is the overpotential, α is the transfer coefficient, T is the temperature, R is the gas constant (8.314 J mol^{-1} K^{-1}), i_k is the kinetic current density normalized to the ECSA, and F is the Faraday constant (96485 C/mol).

Preparation of Pt film electrode for SEIRAS

Pt film electrodes were prepared based on previously reported deposition procedure[1]. The Si prism was first polished with 0.05 μm alumina slurry until the surface became totally hydrophobic. The polished Si prism was then ultrasonicated in water for 10 min and then rinsed with acetone and milli-Q water for multiple times. Subsequently, the Si prism was immersed in NH_4F (40%) for 360 s to obtain a hydrogen-terminated surface, after which the Si prism was immediately immersed in a mixture of 2 wt% aqueous HF solution and Au plating solution with a ratio of 1:4 at around 55 °C for 6 min to deposit an Au film on prism. The Au plating solution was prepared by mixing 5.75 mM $NaAuCl_4 \cdot 2H_2O$ (99%), 0.025 M NH_4Cl (99.99%), 0.025 M $Na_2S_2O_3 \cdot 5H_2O$ (98%), 0.075 M Na_2SO_3 (98%), and 0.026 M NaOH (99%), all of which were purchased from Sigma-Aldrich. The Au-coated prism was used as the working electrode, on which Pt was electrodeposited in Ar saturated 4 mM H_2PtCl_6 + 0.7 M Na_2HPO_4 solution. The electrodeposition current density and time were set at -400 uA cm^{-2} and 360 s, respectively. The prepared Pt film was cleaned with water several times and used immediately.

In situ SEIRAS studies

The SEIRAS experiments were performed in a two-compartment spectroelectrochemical flow cell separated by a piece of anion exchange membrane. The Pt thin film electrode deposited on the Si prism was employed as the working

electrode. A graphite rod was used as the counter electrode. The electrolytes used in all spectroelectrochemical experiments were prepared by the same method as for electrochemical testing in the previous section. SEIRAS experiments were conducted on a Bruker INVENIO S FTIR spectrometer equipped with a liquid nitrogen-cooled MCT detector and a ATR accessory. A Bio-logic sp150e Potentiostat was used to control the electrode potential. An Ag/AgCl electrode (3.0 M KCl, BASi) was used as the reference electrode. All spectra were 64 co-added scans with 4 cm^{-1} resolution.

Electrochemical impedance spectroscopy

EIS experiments were performed with a peak-to-peak amplitude of 5mV ranging from 10 kHz to 0.1Hz. The spectra were fitted to the equivalent electric circuit (EEC) as shown in **Fig. S8** by the software Gamry Echem Analyst using the Levenberg–Marquardt Method for nonlinear least-squares regression.

Molecular dynamics simulations

Atomistic molecular dynamics (MD) simulations were carried out to study the hydration properties of single ions and the properties of electrolytes at the Pt-aqueous interface. Structure model was produced by XPONGE[2].

For the system containing the Pt slab, the square slab consists of three layers of Pt(111) with an area of 30.932 × 29.223 Å^2 (11×12 Pt atoms per layer). An aqueous solution system with 2100 water molecules, 20 cations and hydroxides was constructed above the Pt slab plane based on the surface coverage of the experiment, i.e., the reduction of the H_{upd} feature in CVs. Vacuum was placed above the bulk solution.

To study the properties of the hydration layer of free cations, for each type of cations, a single cation system was built. Each system consists of 5000-water cubic solvent box and one cation placed in the center of the box.

MD simulations were performed by the SPONGE[3] program and the force field parameters for TMA/TEA/TPA and hydroxide were taken from general AMBER force field[4]. The atomic point charges were calculated by Multiwfn using RESP charge model[5,6]. The TIP4P-EW model was chosen for water molecules[7]. The Lennard–Jones parameters for K^+ and Pt were taken from the literature[8,9]. 10000-step gradient descent minimization was carried out before the systems were simulated. First, a 10 ns pre-equilibrium was carried out with the time step of 2 fs, in which the temperature of the system was set to 300 K by the middle scheme of Langevin dynamics[10]. For the single cation system, an additional 2 ns pressure-equilibrium step before pre-equilibrium step was carried out using NPT ensemble with the time step of 2 fs, in which the temperature

of the system was set to 300 K by the middle scheme of Langevin dynamics and the pressure of the system was set to 1 atm using Monte-Carlo barostat[11]. The final production simulations were simulated for 30 ns in the canonical ensemble with the time step of 1 fs, in which the temperature of the system was set to 300 K by the middle scheme of Langevin dynamics. For the system with a Pt slab, the electric potential of the top surface was limited to -0.87 V relative to vacuum by FEM module in SPONGE[3]. Specifically, finite element grids are first initialized, after which the electric potential distribution is obtained by solving Poisson's equation under a given boundary condition (In our work, the boundary condition is ϕ=-0.87 V on the Pt surface and ϕ=0 V in the vacuum). The simulation with Pt slab was done in two dimensions with periodic boundary and double-Layer equipotential boundary conditions(The Pt slab system is periodic in the X & Y directions and has two equipotential boundary in the Z direction). The simulation trajectories were visualized by VMD software[12,13].

Determination of hydrogen bond lifetimes

Identifying hydrogen bonds and calculating their lifetimes were performed using the Python package MDAnalysis[14,15,16]. hydrogen bond lifetime was calculated via the time autocorrelation function of the presence of a hydrogen bond:

$$C(\tau) = \left\langle \frac{h_{ij}(t_0)h_{ij}(t_0+\tau)}{h_{ij}(t_0)^2} \right\rangle$$

where the value of h_{ij} represents whether there is a hydrogen bond between atoms i and j. $h_{ij} = 1$ means that there is a hydrogen bond between atoms i and j while $h_{ij} = 0$ means there is no hydrogen bond between these two atoms. Criteria for identifying

hydrogen bonds are: 1) the donor-acceptor distance is ≤ 3 Å, and 2) the donor-hydrogen-acceptor angle is ≤ 150°. The average lifetime of the hydrogen bond was obtained by integrating the autocorrelation function:

$$lifetime\ of\ hydrogen\ bond = \int_0^{\infty} C(\tau)d\tau$$

Statistical length distribution of hydrogen bond chains

In order to count the length distribution of hydrogen-bonded water chains at the Pt(111)-organic cations interface, we selected the hydrogen-bonded water chains between the water with distance from the surface (z) < 4.4 Å (surface adsorbed water layer) and the z > 10.4 Å. We first counted water with z < 12 Å (slightly greater than 10.4 Å in order to identify water molecules located at just outside the cutoff distance of 10.4 Å from the surface) and total hydrogen bonds formed among these water molecules. Then we chose water molecules with z < 4.4 Å and z > 10.4 Å as the starting and ending points, respectively (vice versa). The horizontal distance between the starting and ending points was constrained within 3 Å. The shortest connecting path between the starting and ending points was was found by Floyd algorithm and selected as the length of the hydrogen bond chain for statistics.

Supplementary Note I: Potential effect of impurities in the electrolytes

It is well known that impurities in the electrolyte could significantly influence the results of electrochemical measurements[17-18]. Due to the difficulty in chemical purify the commercial tetraalkylammonium hydroxide solutions via common techniques such as recrystalization, these electrolytes were used without further treatment. Characterizations and control expeirments were conducted to ensure that the measured CV features and HOR/HER activities were not impacted by the impurities in the electrolyte. No impurities could be identified in the ^{1}H NMR spectra of all tetraalkylammonium cations employed in this work (Fig. S1), suggesting the low level of decomposition of organic cations and low concentrations of other organic contaminations. The H_{upd} features were suppressed in 0.1 M TPAOH (Fig. 1b in the main text), and the H_{upd} features were largely recovered when the electrolyte was switched to 0.05 M TPAOH + 0.05 M KOH on the same electrode surface (without any treatment in between, Fig. S2). The clear electrolyte dependence of the H_{upd} features indicates that no impurities associated with TPAOH permenantly contaminates the Pt surface. In addition, 0.1 M TPAOH was preelectrolyzed at 0.01 V for 8500 s with a Pt electrode to remove any impurities that could deposit on the Pt during reaction before collecting CVs and determining the HOR/HER activities on a fresh Pt electrode (Fig. S3a). The CVs collected in 0.1 M TPAOH with and without the preelectrolysis treatment overlap (Fig. S3b), confirming that no detectable level of depositable impurities is present in the electrolyte. 50 consecutive CV scans of Pt in 0.1 M TPAOH also overlap (except for the first scan), suggesting negligible decomposition of the

electrolyte (Fig. S4). No appreciable difference was observed between polarization curves on Pt collected in 0.1 M TPAOH with and without the preelectrolysis treatment (Fig. S5), suggesting that no contaminants in the TPAOH electrolyte affect the measured activities.

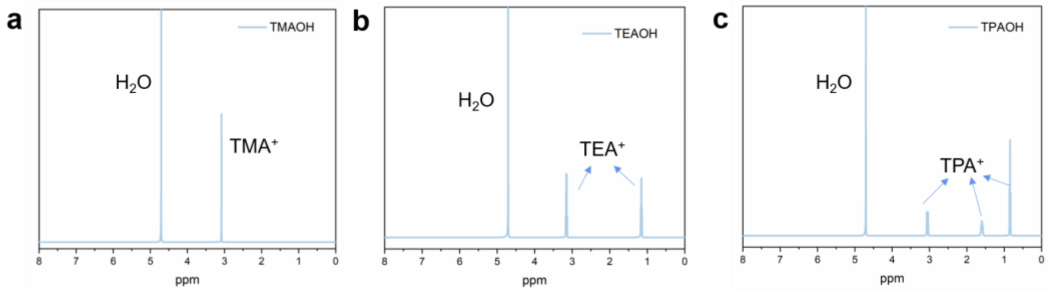

Fig. S1. ^{1}H NMR spectra of the solutions of TMAOH, TEAOH and TPAOH.

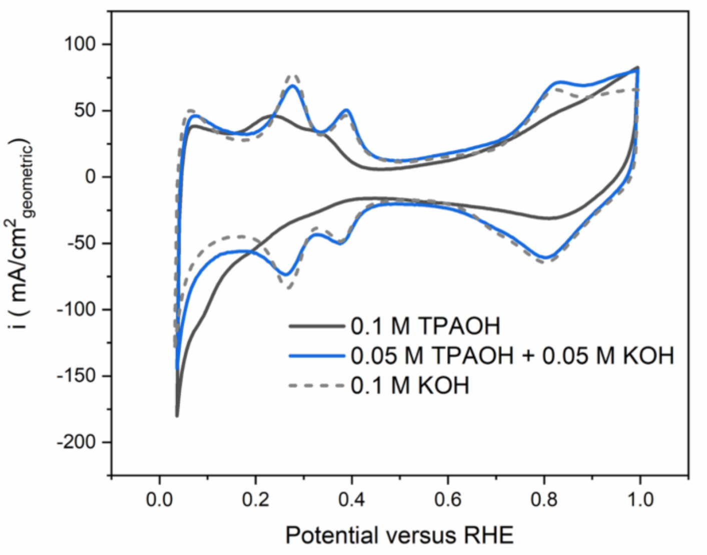

Fig. S2. CV curve on pc Pt at 50 mV/s in Ar saturated 0.1 M TPAOH (black trace), and the electrolyte was switched to 0.05 M TPAOH + 0.05 M KOH (on the same Pt electrode without any cleaning) before the CV curve was collected (blue trace). CV curve in 0.1 M KOH (light grey race) collected on a fresh pc Pt electrode is included for comparison.

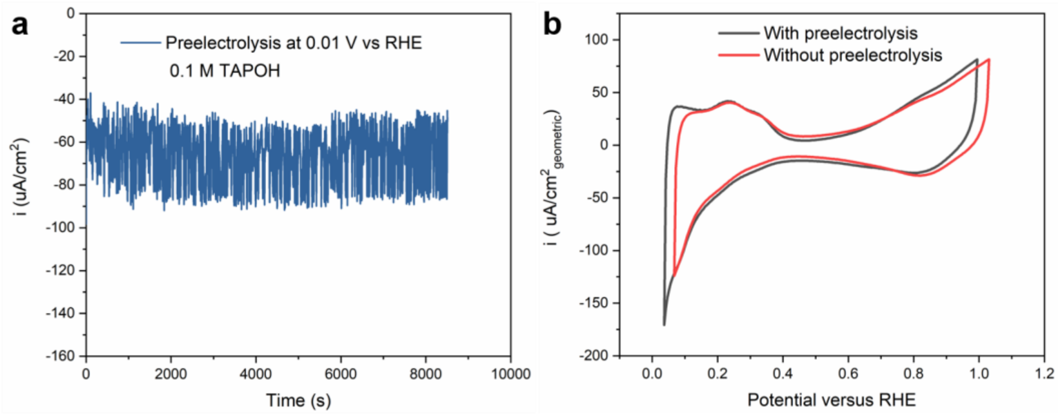

Fig. S3. (**a**) Preelectrolysis at 0.01 V vs RHE in 0.1 M TPAOH for 8500 s. (**b**) The CV curves collected in 0.1 M TPAOH with and without the preelectrolysis.

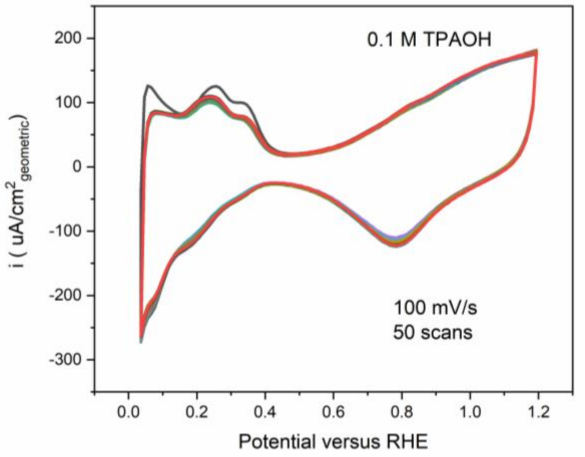

Fig. S4. 50 CV scans in the range of 0.05-1.2 V to remove any reducing species. Except for the the first CV scan (black trace), the rest of scans essentially overlap.

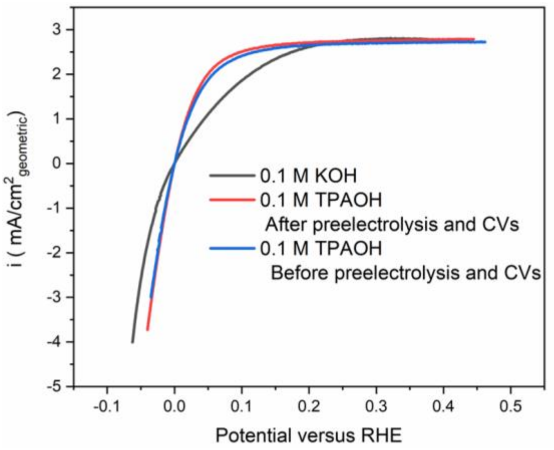

Fig. S5. HOR/HER polarization curve on pc Pt in H_2 saturated 0.1 M TPAOH with and without preelectrolysis.

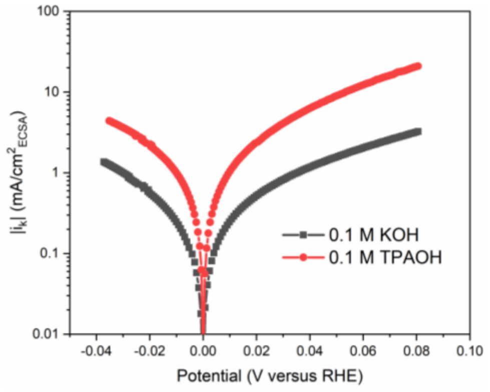

Fig. S6. Measured and fitted (with the Butler–Volmer equation) HOR/HER kinetic current densities on pc-Pt in 0.1 M KOH and 0.1 M TPAOH at 10 mV/s.

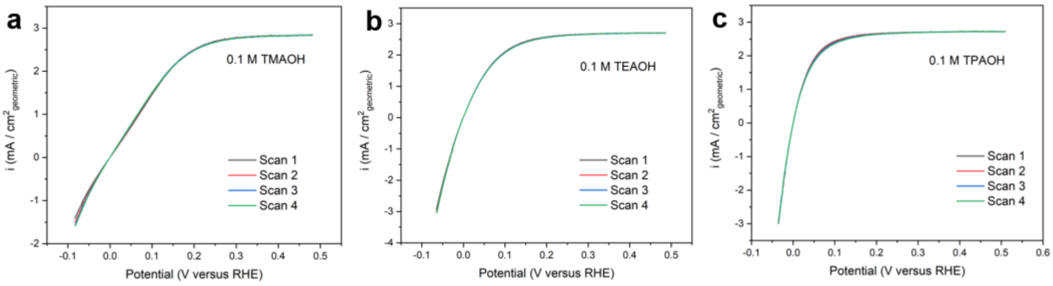

Fig. S7. HOR/HER polarization curves of multiple scans on pc-Pt in H_2 saturated (**a**) 0.1 M TMAOH, (**b**) 0.1 M TEAOH and (**c**) 0.1 M TPAOH.

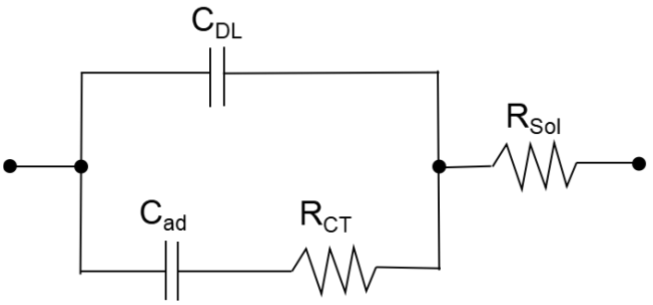

Fig. S8. Equivalent electric circuit for the H_{upd} region. R_{sol} represents the resistance of solution, while C_{DL} and C_{ad} stand for electric double layer capacitance and the pseudo-capacitance of the adsorbed hydrogen intermediate, respectively.

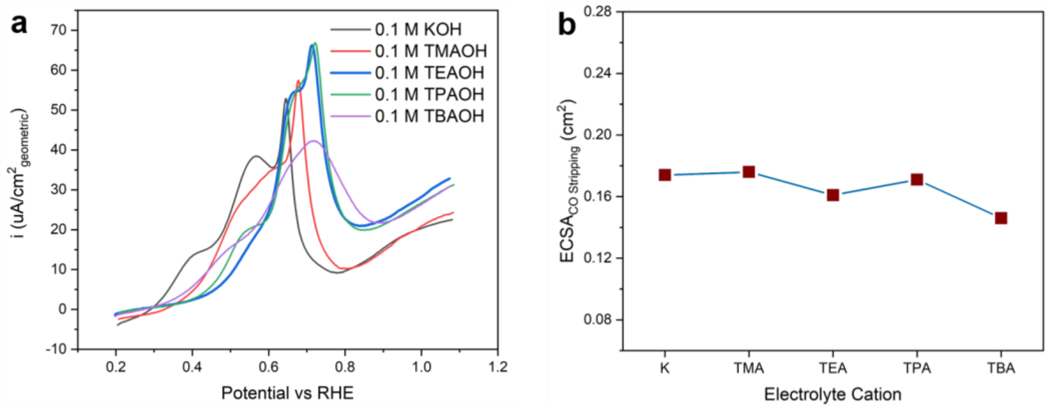

Fig. S9. (**a**), CO stripping plots on pc-Pt in 0.1 M hydroxide solution with different cations. The scanning rate is 20 mV/s. (**b**), Integrated ECSA based on the charge of CO oxidation.

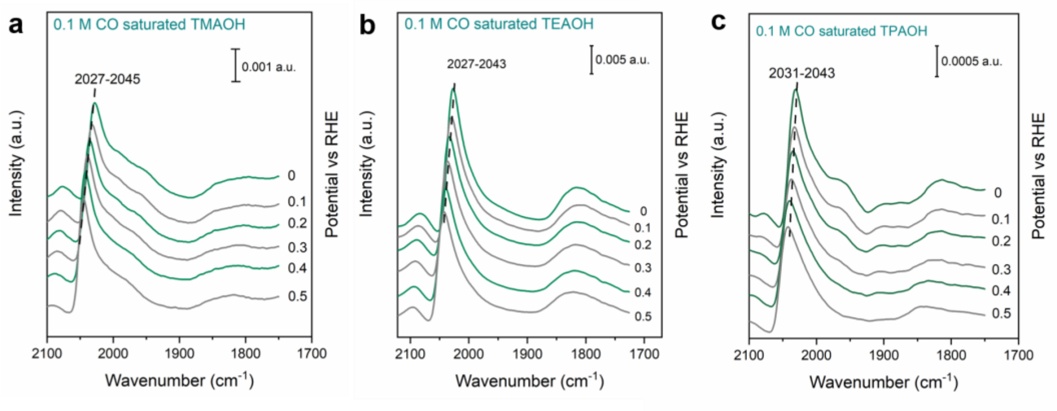

Fig. S10. The potential dependent IR spectra for CO adsorption in the solutions of organic cations.

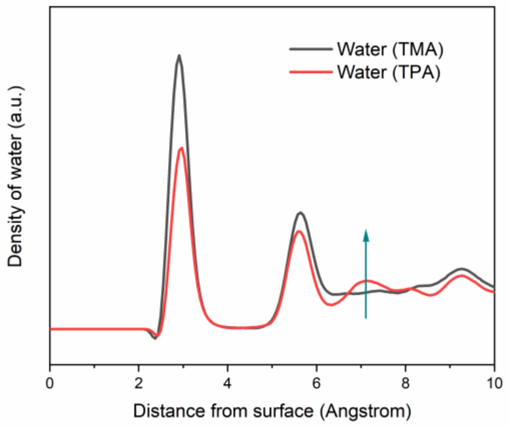

Fig. S11. Distribution functions of water molecules on Pt (100) surface with TMA and TPA as electrolyte cation along the direction of surface normal.

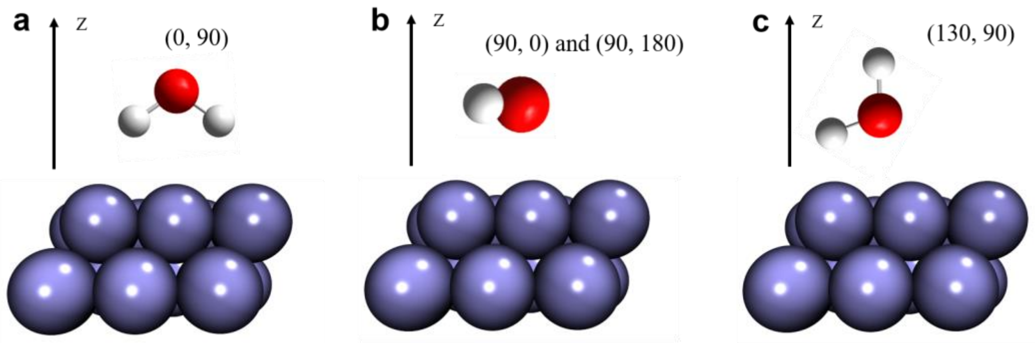

Fig. S12. Schematic of water orientations for **a,** (0,90), **b,** (90,0) and (90,180), and **c,** (130, 90).

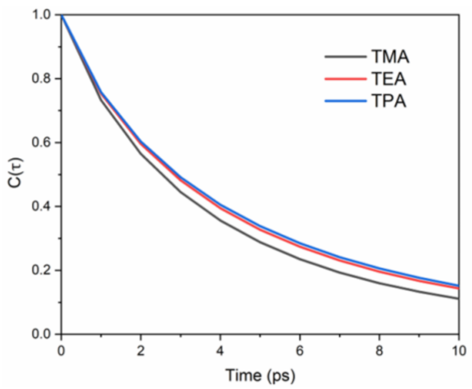

Fig. S13. The time autocorrelation function of hydrogen bonds in the MD simulations with TMA, TEA and TPA.

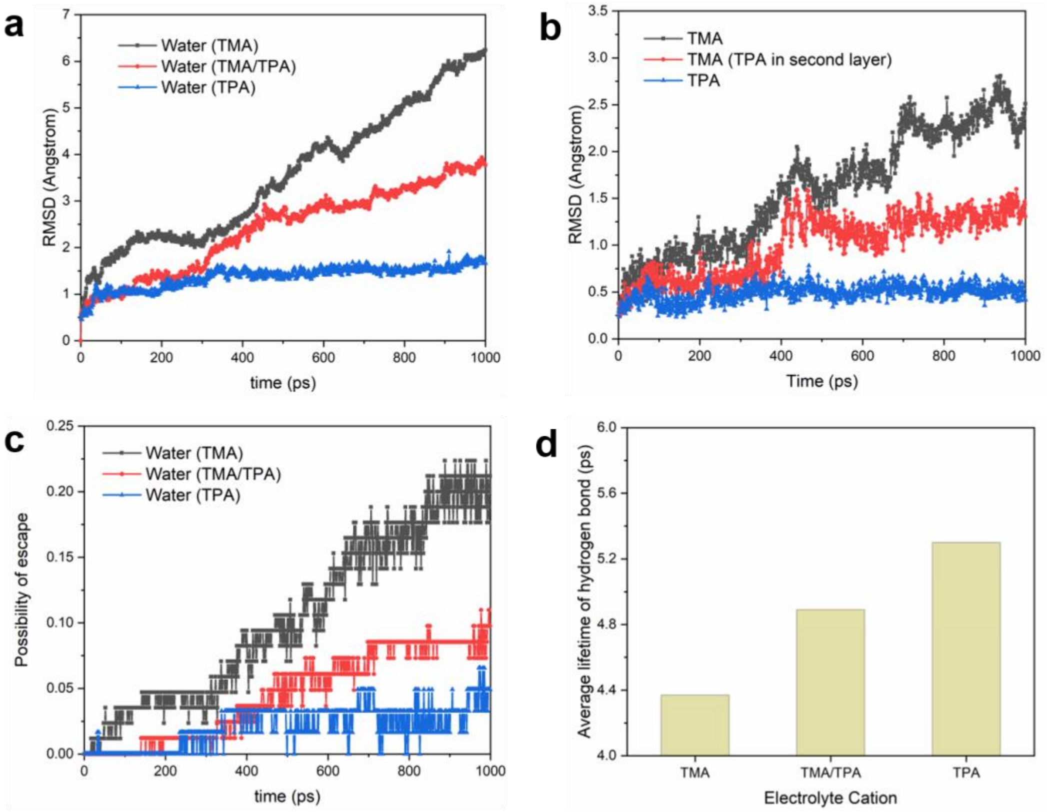

Fig. S14. (**a**). Root-mean-square displacement of water in the TMA/TPA mixed system. (**b**). Root-mean-square displacement of TMA in the TMA/TPA mixed system. (**c**). Possibility of escape of water from Pt(111) surface in the TMA/TPA mixed system. (**d**). Extracted lifetime of hydrogen bonds from time autocorrelation functions in the TMA/TPA mixed system.

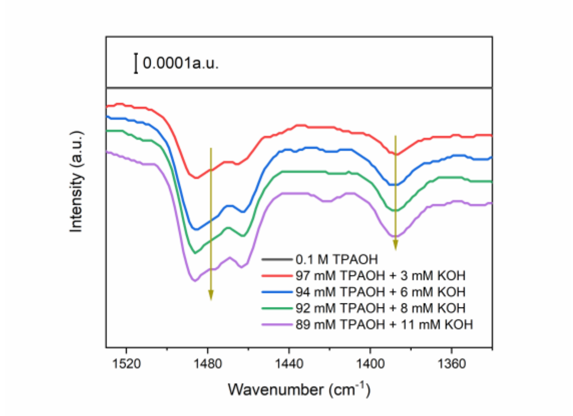

Fig. S15. SEIRA spectra of C-H bending modes in Ar saturated 0.1 M hydroxide solution with various ratios of TPA and K^+. The reference spectrum was obtained in 0.1 M TPAOH.

Table S1. Average number of hydrogen bonds of water molecules surrounding single cation

Cation	K	TMA	TPA
Average number of hydrogen bond	2.4	3.0	3.1

Table S2. The pH values of the electrolytes of organic cations used in this work.

Electrolyte	pH
0.1 M TMAOH	12.96
0.1 M TEAOH	13.03
0.1 M TPAOH	13.01

References

1 Zhu, S., Qin, X., Yao, Y. & Shao, M. pH-Dependent Hydrogen and Water Binding Energies on Platinum Surfaces as Directly Probed through Surface-Enhanced Infrared Absorption Spectroscopy. *J. Am. Chem. Soc.* **142**, 8748-8754, (2020).

2 Xia, Y. & Gao, Y. Q. Xponge: A Python package to perform pre- and post-processing of molecu lar simulations. *Journal of Open Source Software* **7**, 4467, (2022).

3 Huang, Y.-P. *et al.* SPONGE: A GPU-Accelerated Molecular Dynamics Package with Enhanced Sampling and AI-Driven Algorithms. *Chin. J. Chem.* **40**, 160-168, (2022).

4 Wang, J., Wolf, R. M., Caldwell, J. W., Kollman, P. A. & Case, D. A. Development and testing of a general amber force field. *J. Comput. Chem.* **25**, 1157-1174, (2004).

5 Lu, T. & Chen, F. Multiwfn: A multifunctional wavefunction analyzer. *J. Comput. Chem.* **33**, 580-592, (2012).

6 Zhang, J. & Lu, T. Efficient evaluation of electrostatic potential with computerized optimized code. *Phys. Chem. Chem. Phys.* **23**, 20323-20328, (2021).

7 Horn, H. W. *et al.* Development of an improved four-site water model for biomolecular simulations: TIP4P-Ew. *J. Chem. Phys.* **120**, 9665-9678, (2004).

8 Li, P., Song, L. F. & Merz, K. M., Jr. Systematic Parameterization of Monovalent Ions Employing the Nonbonded Model. *J. Chem. Theory Comput.* **11**, 1645-1657, (2015).

9 Heinz, H., Vaia, R. A., Farmer, B. L. & Naik, R. R. Accurate Simulation of Surfaces and Interfaces of Face-Centered Cubic Metals Using 12−6 and 9−6 Lennard-Jones Potentials. *J. Phys. Chem. C* **112**, 17281-17290, (2008).

10 Zhang, Z. *et al.* A unified thermostat scheme for efficient configurational sampling for classical/quantum canonical ensembles via molecular dynamics. *J. Chem. Phys.* **147**, 034109, (2017).

11 Åqvist, J., Wennerström, P., Nervall, M., Bjelic, S. & Brandsdal, B. O. Molecular dynamics simulations of water and biomolecules with a Monte Carlo constant pressure algorithm. Chemical Physics Letters 384, 288-294, (2004).

12 Humphrey, W., Dalke, A. & Schulten, K. VMD: Visual molecular dynamics. *J. Mol. Graphics* **14**, 33-38, (1996).

13 Berendsen, H. J. C., Postma, J. P. M., van Gunsteren, W. F., DiNola, A. & Haak, J. R. Molecular dynamics with coupling to an external bath. *J. Chem. Phys.* **81**, 3684-3690, (1984).

14 Michaud-Agrawal, N., Denning, E. J., Woolf, T. B. & Beckstein, O. MDAnalysis: A toolkit for the analysis of molecular dynamics simulations. *J. Comput. Chem.* **32**, 2319-2327, (2011).

15 Gowers, R. J. *et al*. MDAnalysis: A Python package for the rapid analysis of molecular dynamics simulations. *Proceedings of the 15th Python in Science Conference* (eds Sebastian Benthall & Scott Rostrup) 98-105 (2016).

16 Smith, P., Ziolek, R. M., Gazzarrini, E., Owen, D. M. & Lorenz, C. D. On the interaction of hyaluronic acid with synovial fluid lipid membranes. *Phys. Chem. Chem. Phys.* **21**, 9845-9857, (2019).

17. M. Dunwell, X. Yang, Y. Yan, B. Xu, Potential Routes and Mitigation Strategies for Contamination in Interfacial Specific Infrared Spectroelectrochemical Studies. J. Phys. Chem. C **122**, 24658-24664 (2018).

18. W. Sheng, H. A. Gasteiger, Y. Shao-Horn, Hydrogen Oxidation and Evolution Reaction Kinetics

on Platinum: Acid vs Alkaline Electrolytes. J. Electrochem. Soc. **157**, B1529, (2010).
